# Modeling non-uniformity in short-read rates in RNA-Seq data

**DOI:** 10.1186/gb-2010-11-5-r50

**Published:** 2010-05-11

**Authors:** Jun Li, Hui Jiang, Wing Hung Wong

**Affiliations:** 1 Department of Statistics, Stanford University, Sequoia Hall, 390 Serra Mall, Stanford, CA 94305, USA; 2 Stanford Genome Technology Center, 855 California Ave, Palo Alto, CA 94304, USA; 3 Department of Health Research and Policy, Stanford University, 259 Campus Drive, Redwood Building, Stanford, CA 94305, USA

## Abstract

After mapping, RNA-Seq data can be summarized by a sequence of read counts commonly modeled as Poisson variables with constant rates along each transcript, which actually fit data poorly. We suggest using variable rates for different positions, and propose two models to predict these rates based on local sequences. These models explain more than 50% of the variations and can lead to improved estimates of gene and isoform expressions for both Illumina and Applied Biosystems data.

## Abstract 2

Methods for modeling read counts from short read RNA-seq data.

## Background

Microarrays are an efficient technology to measure the expression levels of many genes simultaneously, but there are some limitations to this method. The expression estimates are typically not reliable for lowly expressed genes because the true signals are masked by cross-hybridization effects [1,2]. Furthermore, the design of the array depends on annotation of gene structures and thus the method is not ideal for the discovery of novel splicing events. A recently developed alternative approach, called RNA-Seq, has the potential to overcome these difficulties [3]. RNA-Seq uses ultra-high-throughput sequencing [4] to determine the sequence of a large number of cDNA fragments. The resulting sequences (reads) can be long (>100 nucleotides) or short, depending on the platform [4]. Two currently popular short-read platforms are Illumina's Solexa [5-11] and Applied Biosystems' (ABI's) SOLiD [12]. Each can produce tens of millions of short reads in a single run [5-12]. In this paper, we only consider the short-read RNA-Seq.

The reads produced by RNA-Seq are first mapped to the genome and/or to the reference transcripts using computer programs. Then, the output of RNA-Seq can be summarized by a sequence of 'counts'. That is, for each position in the genome or on a putative transcript, it gives a count standing for the number of reads whose mapping starts at that position. As an example (we have shortened the gene and reads for simplification), if a gene with a single isoform has sequence ACGTCCCC, and we have 12 ACGTC reads, 8 CGTCC reads, 9 GTCCC reads, and 5 TCCCC reads, then this gene can be summarized by a sequence of counts 12, 8, 9, 5.

Quantitative inference of RNA-Seq data, such as calculating gene expression levels [7] and isoform expression levels [13], is based on these counts. To utilize the data efficiently, it is crucial to have an appropriate statistical model for these counts. Current analysis methods assume, explicitly or implicitly, a naive constant-rate Poisson model, in which all counts from the same isoform are independently sampled from a Poisson distribution with a single rate proportional to the expression level of the isoform [7,13,14]. Unfortunately, we found that this model does not provide a good fit to real data (see Results), and a more elaborate model is needed.

To better model the counts, it is natural to consider a Poisson model with variable rates; that is, the counts from an isoform are still modeled as Poisson random variables, but each Poisson random variable has a different rate (mean value). By checking the similarities among counts of different tissues (see Results), one can see that the Poisson rate depends on not only the gene expression level, but also the position of the read. Hence, we model the rate as the product of the gene expression level and the 'sequencing preference' of reads starting at this position. This sequencing preference is a factor showing how likely it is for a read to be generated at this position.

Dohm *et al. *[15] found that GC-rich regions tend to have more reads than AT-rich regions, but we find that models based purely on GC content work poorly (Additional file 1). Some clues on how to model the sequencing preferences may be obtained by reviewing how related issues are handled in microarrays. There are a set of probes for each gene in microarrays, and each probe gives a continuous measurement of the gene expression level. The values of the measurements from the same set are modeled by a Gaussian distribution with different means, each of which is the product of the gene expression level and the affinity of that probe to the cDNA sequences. Naef and Magnasco [16] proposed a model for the probe affinities, which only depends on the probe sequences:

where *ω*_*i *_is the affinity of probe *i*, *K *is the length of the probe, I(*b*_*ik *_= *h*)) is 1 when the *k*^*th *^base pair is letter *h*, and 0 otherwise, *α *and *β*_*kh *_are the parameters we want to estimate, and *ε *is Gaussian noise so that the parameters can be estimated by regular linear least squares. The key feature of this model is that it considers the letter appearing at each location, rather than just the total number of occurrences of each letter. This simple linear model can explain 44% of the differences of the affinities in an Affymetrix oligonuleotide array dataset. Similar models have been developed for other arrays or datasets [17-20].

In RNA-Seq experiments, cDNA synthesis is typically initiated by random priming. Depending on its sequence, an mRNA fragment may form secondary structures that obstruct the binding of the primers. Furthermore, the primer is usually tagged by a non-random flanking sequence that may preferentially interact with the mRNA depending on the mRNA sequence. Due to these effects, the probability for binding depends on both the nucleotide sequence and the protocol. After synthesis, the cDNAs are ligated to linkers, amplified and then sequenced. In these steps, the secondary structure of the cDNA and the details of the protocol can again influence the efficiency. Therefore, the protocol and the local sequence context may have a large influence on how likely an mRNA segment will be read. Hence, under a specific protocol, we may be able to predict, at least partly, the sequencing preferences based on the local nucleotide sequences.

## Results and discussion

### Datasets and overdispersion

Three genome-wide RNA-Seq datasets are used in this paper. The first two were generated by Illumina's Solexa platform, and the third one was generated by ABI's SOLiD platform. The first dataset [7] is composed of 79, 76, and 70 million reads from three mouse tissues: brain, liver and skeletal muscle. Each read is of length 25. The second dataset [11] is composed of 12 to 29 million reads from 10 diverse human tissues and 5 mammary epithelial or breast cancer cell lines. Each read is of length 32. We use data from nine of these tissues or cell lines, and merge them into three groups (adipose, brain, and breast in group one, colon, heart, and liver in group two, lymph node, skeletal muscle, and testes in group three.). Each group contains 61 to 77 million reads. The third dataset [12] is composed of 16 million high-quality reads from each of the two cell lines: embryoid bodies (EB) and undifferentiated mouse embryonic stem cells (ES). Each original read is 35 nucleotides, but some are truncated into 30 or 25 nucleotides to ensure high quality. We refer to these three datasets as Wold data, Burge data, and Grimmond data, respectively, in accord with the research group that originally generated the data. As we just described, each of the three datasets contains several sub-datasets standing for different tissues, groups, or cell lines, and in total we have eight sub-datasets: three (tissues) for Wold data, three (groups) for Burge data, and two (cell lines) for Grimmond data. In all our processing and calculations, the above sub-datasets are considered separately; that is, only one sub-dataset is analyzed at a time.

First, the count data are extracted from the original datasets. The detailed procedure is described in Materials and methods. Briefly speaking, we map reads to all isoforms of all RefSeq genes, and then in order to avoid ambiguity, we only count reads uniquely mapped to genes that have only one isoform annotated in RefSeq and do not overlap with other genes, which we call 'non-overlapped single-transcript genes'. Further, we use only the counts from the top 100 genes with the highest expression levels to fit our model since they have the highest signal-to-noise ratio (see Additional file 1 for details).

**Figure 1:**
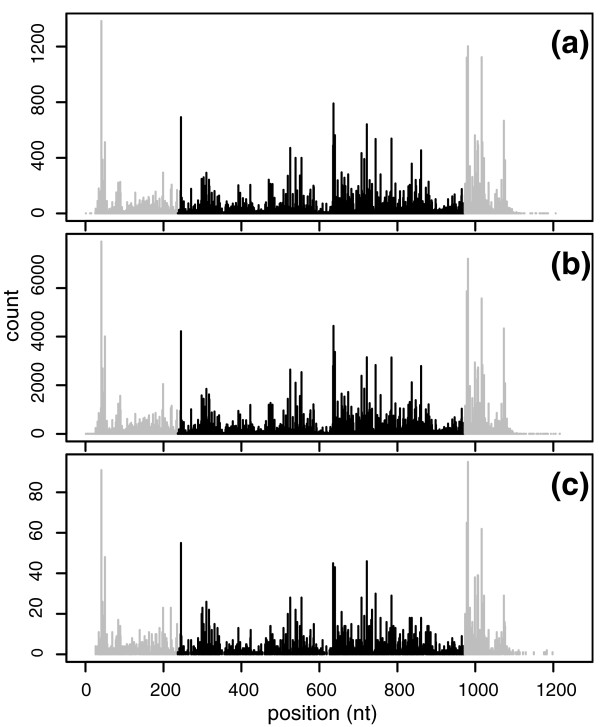
**Counts of reads along gene *Apoe *in different tissues of the Wold data**. (**a**) Brain, (**b**) liver, (**c**) skeletal muscle. Each vertical line stands for the count of reads starting at that position. The grey lines are counts in the UTR regions and a further 100 bp. Here introns are deleted and exons are connected into a single piece. Only shown are counts on one strand of the gene; counts on the other strand show similar similarities in different tissues. Nt: nucleotides.

Two pieces of evidence clearly show that the counts violate the Poisson model with a constant rate. First, the data are seriously overdispersed. A basic property of Poisson distribution is the equality of mean and variance. If variance is larger than mean, then the data are said to be overdispersed, and the Poisson assumption is inappropriate. Table 1 lists the maximum, median, and minimum values of the variance-to-mean ratios (also called 'Fano factor') in the top 100 genes of each sub-dataset. All the ratios are much larger than 1. Second, the 'pattern' (relative values) of counts across a gene is surprisingly conserved in different sub-datasets of the same dataset. Figure 1 shows the counts in the gene *Apoe *(apolipoprotein E) of all three tissues of the Wold data. Although the absolute values of the counts varies by 100-fold in different tissues, the patterns of variation are highly consistent across tissues. The same holds true in other genes of the Wold data and in genes of the Burge and Grimmond data. This is strong evidence that the counts for different positions from the same gene are not sampled from the same distribution. Rather, the distribution of a count seems to depend on the position of its sequence in the transcript. This compels us to consider more sophisticated models. The observation that the biases in read rates are strongly dependent on local sequences has also been described by Hansen *et al. *[21], which is an independent work that came to our attention when our paper was under review.

**Table 1:** Variance-to-mean ratios in different datasets

		Variance-to-mean ratios		
		
Dataset	Sub-dataset	Maximum	Median	Minimum
Wold	Brain	248	36	21
	Liver	1,503	48	19
	Muscle	2,088	34	18
				
Burge	Group 1	835	78	14
	Group 2	1,187	102	28
	Group 3	1,593	112	20
				
Grimmond	EB	24,385	806	47
	ES	9,162	345	22

### The Poisson linear model and its performance

For nucleotide *j *of gene *i*, we want to model how the distribution of the count of reads starting at this nucleotide (denoted as *n*_*ij*_) depends on the expression level of this gene (denoted as *μ*_2_) and the nucleotide sequence surrounding this nucleotide (the sequence with length *K *is denoted as *b*_*ij*1_, *b*_*ij*2_, ⋯, *b*_*ijK*_,). We assume *n*_*ij*_~Poisson (*μ*_*ij*_), where *μ*_*ij *_is the rate of the Poisson distribution, and *μ*_*ij *_= *ω*_*ij *_*μ*_*ij*_, where *ω*_*ij *_is the sequencing preference, which may depend on the surrounding sequence. As a simple approach, we use a linear model for the preference and hence the Poisson rate:

where *ν*_*I *_= log(*μ*_*i*_), *α *is a constant term, I(*b*_*ijk *_= *h*) equals to 1 if the *k*^*th *^nucleotide of the surrounding sequence is *h*, and 0 otherwise, and *β*_*kh *_is the coefficient of the effect of letter *h *occurring in the *k*^*th *^position. This model uses about 3K parameters to model the sequencing preference. To fit the above model, we iteratively optimize the gene expression levels and the Poisson regression coefficients (Materials and methods).

We applied our model to each of the eight sub-datasets. As local sequence context, we use 40 nucleotides prior to the first nucleotide of the reads and 40 nucleotides after them (that is, the first 40 nucleotides of the reads; see Additional file 1 for the reason for choosing this region). Thus, our model uses 3 × 80 = 240 parameters to model the sequencing preference. This is a relatively small number compared to the sample size (about 100,000 counts) in each sub-dataset.

In linear regression, the percentage of variance that can be explained by the regression, denoted by *R*^2^, is used to measure the goodness-of-fit. In Poisson regression, we can replace variance by deviance and define:

where *d *is the deviance of the fitted model, and *d*_0 _is the deviance of the null model [22]. In our case, the null model is the naive model assuming the same sequencing preference. The final *R*^2 ^values we achieved are listed in Table 2. Roughly speaking, this simple linear model can explain about 40 to 50% of the variance.

**Table 2:** *R*^2 ^in different datasets

		*R* ^2^			
		
		Poisson linear			MART
			
Dataset	Sub-dataset	80 nucleotides^a^, non-cross-validation	80 nucleotides^a^, cross-validation	40 nucleotides^a^, cross-validation	40 nucleotides^a^, cross-validation
Wold	Brain	0.52	0.51	0.51	0.70
	Liver	0.51	0.50	0.50	0.70
	Muscle	0.48	0.46	0.46	0.59
					
Burge	Group 1	0.43	0.42	0.42	0.52
	Group 2	0.37	0.35	0.35	0.46
	Group 3	0.45	0.42	0.42	0.54
					
Grimmond	EB	0.47	0.40	0.40	0.58
	ES	0.45	0.39	0.37	0.54

^a^The lengths of the surrounding sequences we consider.

**Figure 2:**
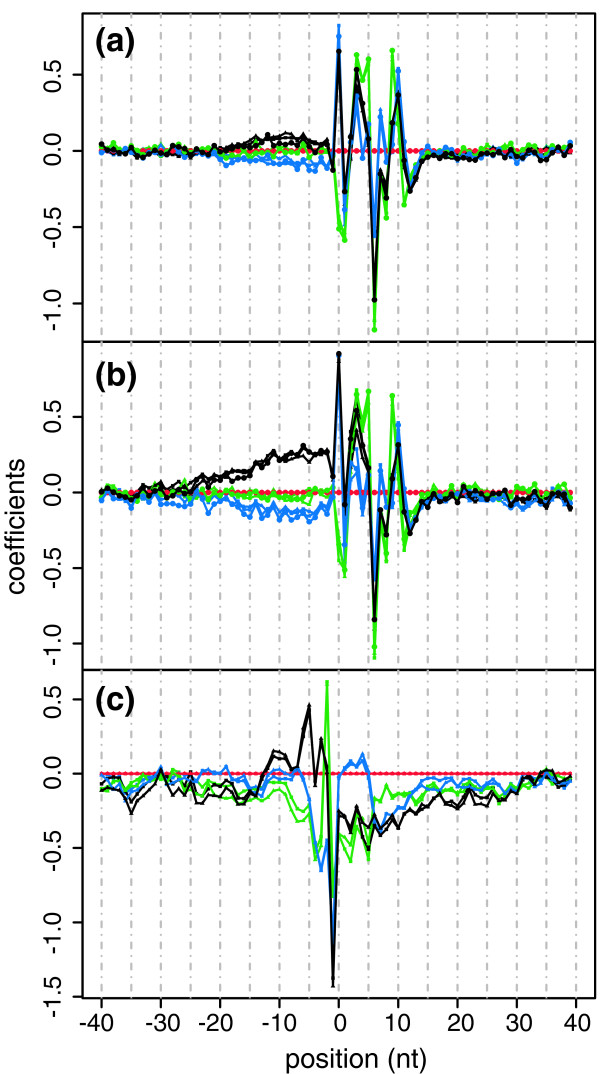
**The coefficients of the Poisson linear models in different datasets**. The coefficients of the Poisson linear model in the eight sub-datasets when we consider surrounding sequences as 40 nucleotides before and 40 nucleotides after the first nucleotide of a read. Position -1, 0, 1 means the nucleotide before the first nucleotide of a read, the first nucleotide of a read, and the second nucleotide of a read, respectively. Color coding for nucleotides: red, T; green, A; blue, C; black, G. The coefficients for nucleotide T (red) are the base levels, so they are always zero. (**a**) Coefficients in the Wold data. Shape coding for sub-datasets: rectangle, brain; triangle, liver; circle, skeletal muscle. (**b**) Coefficients in the Burge data. Shape coding for sub-datasets: rectangle, group 1; triangle, group 2; circle, group 3. (**c**) Coefficients in the Grimmond data. Shape coding for sub-datasets: rectangle, EB; triangle, ES. Following are examples of how these coefficients should be read. In the Wold brain data, the coefficient of C in the first nucleotide of a read (the blue rectangle at position 0 in (a)) is 0.82. This means that if the nucleotide T is replaced by C, then the sequencing preference will increase to *e*^0.82 ^= 2.27 times. Nt: nucleotides.

Figure 2 shows all coefficients in the linear model. The asymptotic standard error of each coefficient is approximately 0.002, so almost all coefficients are statistically very significant. This is not surprising, as our sample size is much bigger than the number of parameters. In this case, what are more important are the magnitudes of the coefficients. Generally, the coefficients in the central part of the figure have larger absolute values than those on both sides, where they approach zero. This shows that the nucleotides around the first position of a read have greater effect on the sequencing preference. This is reasonable, as these nucleotides tend to form with the head of a read local secondary structure, which involves only several nucleotides and is thus easy to predict. Although farther nucleotides may form non-local secondary structure with the head of a read, it is hard to predict the structure since it involves too many nucleotides and may differ dramatically from case to case.

The coefficients are strikingly similar in each sub-dataset of the same dataset, although they significantly differ in different datasets. This is strong evidence that these coefficients are meaningful rather than just random.

Although it is difficult to explain biologically the magnitude of each coefficient, it is possible for us to explain the main differences of coefficients between datasets by the protocols they used. Both the Wold and Burge data were generated by using the Illumina platform, so their curves look similar, especially in the central part. However, the mRNAs were fragmented into approximately 200-nucleotide pieces before cDNA synthesis in the Wold data but not in the Burge data. Shorter pieces of mRNA are less likely to form non-local secondary structure. Therefore, the coefficient curve of the Wold data should have lighter tails. Grimmond's experiment used ABI's platform for sequencing and added quite different linkers to the synthesized cDNA before sequencing, so the whole curve looks quite different from that of the Wold and Burge data.

Our Poisson linear model shows that at least 37 to 52% of the non-uniformity can be explained by the sequence difference. However, this percentage may be an underestimate of the fraction of deviance explainable by local sequence context as the simple linear model cannot capture many other effects. Adding more predictors to the linear model is possible, and in particular adding the dinucleotide composition can considerably improve the fitting (Additional file 1), but we prefer to consider nonlinear models to get a better understanding of how much of the non-uniformity of the counts is systematic bias rather than random noise.

### The MART model and its performance

Having tried methods such as support vector machines and neural networks (Additional file 1), we settled on MART (multiple additive regression trees) as our final choice for a nonlinear model. MART is a gradient tree-boosting algorithm proposed by Friedman [23,24]. One version of MART is available in the 'gbm' package [25] of R [26]. Also, to avoid the over-fitting that commonly occurs for nonlinear models, we use cross-validation and *R*^2 ^in the testing data.

The details on using MART and on estimating cross-validation *R*^2 ^are given in Materials and methods. In this analysis, we use shorter surrounding sequences. For the Wold and Burge data, we use 25 nucleotides prior to the first nucleotide of the reads and 15 nucleotides after it, and for the Grimmond data, we use 15 nucleotides prior and 25 nucleotides after. These are the regions that have large coefficients in the Poisson regression model (Additional file 1). Using shorter surrounding sequences lowers the dimensions of the input data, thus shortening the training time and reducing the chance of over-fitting.

**Figure 3:**
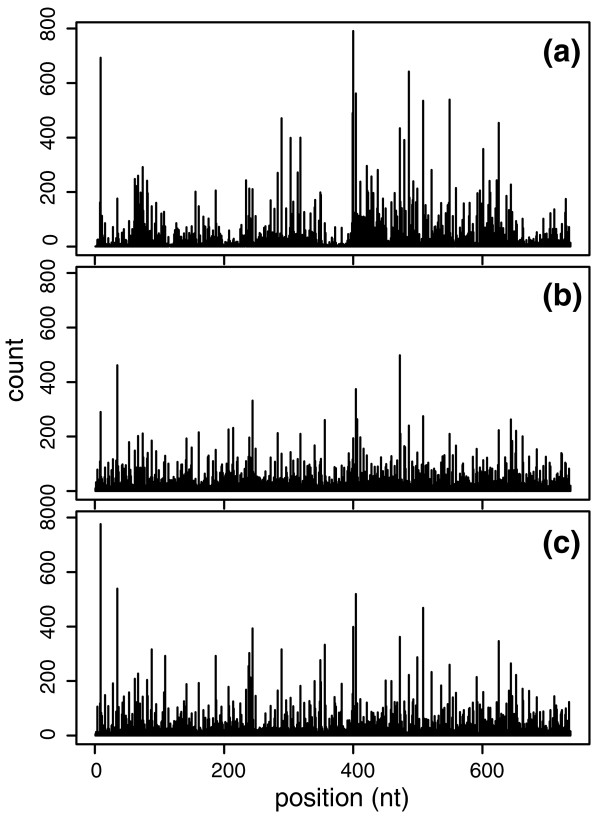
**Fitting counts for the *Apoe *gene**. Black vertical lines represent counts (experimental values or fitted values) along the *Apoe *gene (with the UTR and a further 100 nucleotides truncated). (**a**) Counts of reads (true values) in the Wold brain data. This is the same as the central part (black vertical lines) of Figure 1a. (**b**) Counts of fitted reads using the Poisson linear model. We use the other 99 genes of the top 100 genes to train the linear model, which is then used to predict the counts for *Apoe*. This prediction has a (cross-validation) *R*^2 ^= 0.54. (**c**) Counts of fitted reads using MART. We use the other 99 genes of the top 100 genes to train MART, which is then used to predict the counts for *Apoe*. This prediction has a (cross-validation) *R*^2 ^= 0.69.

The final cross-validation *R*^2 ^values we achieved are listed in Table 2. Seven out of eight *R*^2 ^values are larger than 0.50, and two of them are as high as 0.70. Compared with the linear model, *R*^2^increases by 0.10 to 0.20, showing the power of the MART model. Figure 3 gives us an illustrative example of how our two methods perform. Figure 3a-c shows the counts on gene *Apoe *in the original data, counts fitted by the Poisson linear model, and counts fitted by MART, respectively. It is easy to see that MART fits the counts much better. For this reason, we suggest that the MART model should be used when we make any statistical inferences from the data, while the Poisson linear model is only used to select a reasonable region of surrounding sequences for MART. We also note that the fitted counts determined using MART change more quickly along the gene than those determined using the Poisson linear model, but in neither case are the changes as drastic as in the original data. Actually, the variance-to-mean ratios of fitted counts by the two methods are 55 and 91, both less than 127, the ratio in the original counts. This indicates that both of our models still give conservative fits.

Our high *R*^2 ^shows that at least 50 to 70% of the non-uniformity in the sequencing preference is predictable from local sequences.

**Figure 4:**
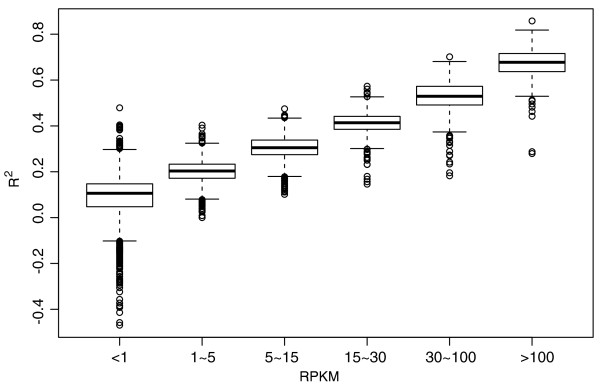
**Boxplot of *R*^2 ^for unique genes in the Wold brain data**. We divided the genes with at least one read into six groups according to their RPKMs: <1, 1 to 5, 5 to 15, 15 to 30, 30 to 100, and >100; each group contains 4,205, 3,320, 2,807, 1,330, 1,094, and 383 genes, respectively. Note that in these data, 1 RPKM stands for 0.034 reads per nucleotide on average, a gene with RPKM >30 is considered to be relatively abundant, and a gene with RPKM <1 is not robust even for transcript detection [7].

The model we trained using the most-highly expressed genes can be used to predict the sequencing preference for other genes. As an example, we predicted for the brain sample of the Wold data the preferences for all unique genes using the MART model trained using the top 100 genes only, and the results are summarized by *R*^2 ^(Figure 4). As expected, *R*^2 ^is smaller for genes with lower expression levels, since unpredictable randomness accounts for a larger portion of variability in a Poisson distribution with a small mean. The average *R*^2 ^is above 0.5 for high or moderately expressed genes (the reads per kilobase of exon per million mapped sequence reads (RPKM) >30), and no *R*^2 ^for genes with RPKM >1 is negative, indicating our model performs consistently better than the uniform model. Note that in these data, 1 RPKM stands for only 0.034 reads per nucleotide on average.

### Applications of our models

Our results may benefit quantitative inference from RNA-Seq data. To reduce biases in gene expression estimates due to non-uniformity of read rates, we propose to estimate the expression of a single-isoform gene by the total number of reads along the gene divided by the sum of sequencing preferences (SSP) under our MART model. In contrast, the standard estimate will divide the number of reads by the length of the gene, which is equivalent to dividing by the SSP under the uniform model where all sequencing preferences are set to be 1.

To test the new method, we first compared the gene expression levels estimated using the mouse liver sub-dataset of the Wold RNA-Seq data with those estimated using Affymetrix microarray data of the same tissue, as used by Kapur *et al. *[27]. For RNA-Seq data, we estimate gene expression level under the uniform model and our MART model, and for microarray data, we use the Robust Multichip Average [28]. All non-overlapped single-transcript genes are included in the comparison, and the results are summarized by the Spearman's rank correlation coefficients. For all genes considered, using our MART model increased the rank correlation from 0.771 to 0.773 compared to the uniform model, which represents a very minor improvement.

What is the reason for the failure of our highly predictive model for sequencing preferences to lead to more significant improvements in gene expression estimates? We believe the answer is that when a gene is large, the dramatic local variations in the sequencing preferences will be smoothed out when they are summed over many positions to produce the SSP for the whole gene. In this case the SSP under the MART model will not be very different from the SSP under the uniform model, and the new estimate will be almost the same as the usual estimate. To see whether the new estimate can lead to improvement in those cases when it is different from the standard estimate, we first quantify the difference between the two estimates by their fold-change, defined as:

The average fold change across genes in the Wold data is only 1.02; thus, it is not surprising that the performance of the new estimate is so close to the standard estimate. Consistently, when we examine the 100 genes with the largest fold changes (on average, the fold change is 1.10 in these 100 genes), the rank correlation shows a much larger improvement, from 0.095 to 0.198, that is, a 108% relative change.

Table 3 presents the average fold changes of genes, exons and junctions of chromosome 1 for the different data sets. We see that the fold change can be substantially larger than 1 depending on how large the region is over which we are averaging the sequencing preferences, the sequencing platform, and the lab that generated the data. For example, the Grimmond data show an average fold change of 1.25 across genes. We thus expect the new estimate will show a greater improvement for this data. To see if this is the case, we note that Kapur *et al. *[27] calculated the gene expression levels of the Affymetrix microarray data from mouse embryo samples, which we can use to assess the new estimate and the standard estimate for the Grimmond EB data. For all genes considered, the rank correlation coefficient increases from 0.439 for the standard estimate to 0.469 for the new estimate, a 6.9% relative change. We further classified the genes into five bins according to their fold change of SSP, each containing about 20% of all genes. Table 4 shows the rank correlation coefficients of gene expression levels for genes in each bin. It is very clear that bigger improvements occur in genes with larger fold changes. For the 20% of genes whose fold changes are the smallest, the improvement is only about 0.1%, but for the 20% of genes whose fold changes are the largest, the improvement is about 26%. Most significantly, for the 100 genes whose fold changes are the largest, the rank correlation changes from 0.323 to 0.526, a 62.8% relative improvement. These results show that our new estimate based on modeling sequencing preferences can lead to significant improvements in gene expression estimates.

**Table 3:** Average fold changes of genes, exons, and junctions of chromosome 1

	Average fold changes of mean sequencing preferences			
	
Dataset used to train the model	Genes	Exons	Junctions (read length = 35)	Junctions (read length = 100)
Wold	1.02	1.12	1.13	1.07
Burge	1.18	1.32	1.37	1.28
Grimmond	1.25	2.17	2.34	1.73

**Table 4:** Spearman's rank correlation coefficients in mouse embryoid bodies

Fold change bin	SCC by uniform model	SCC by our MART model	Relative improvement
(1.00, 1.09)	0.465	0.466	0.1%
(1.09, 1.19)	0.437	0.444	1.4%
(1.19, 1.33)	0.413	0.434	5.1%
(1.33, 1.53)	0.481	0.520	8.2%
(1.53, 4.82)	0.389	0.490	26.0%

SCC: Spearman's rank correlation coefficient.

**Figure 5:**
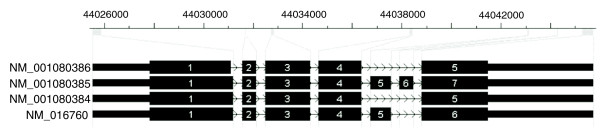
**Four isoforms of RefSeq gene *Clta *in mouse**. This figure was generated using the CisGenome browser [36]. At the top are shown the base positions in mouse chromosome 4 and exons as grey blocks. On the bottom are shown the four isoforms, with exons zoomed in. The tail of exon 1 of the first isoform is 6 bp less than that of the other three isoforms. The second isoform has 7 exons, while the third isoform misses both exon 5 (54 bp) and exon 6 (36 bp), and the fourth isoform misses exon 6.

Next we examined whether incorporation of sequencing preferences can lead to improved inferences for isoform-specific expression levels. We modified the isoform-specific expression estimates in Jiang *et al. *[13] by assuming the mean count for each exon to be proportional to the SSP of the exon instead of the length of the exon. Figure 5 shows the four isoforms of the RefSeq gene *Clta *in mouse. Under the uniform model, the method in [13] gives isoform expression of 21.6%, 53.4%, 8.95%, and 16.0% (let the sum to be 100%) for the Grimmond EB data. When the sequencing preferences are taken into account, the method in [13] gives 15.5%, 52.9%, 10.8%, and 20.7%. The new counts based on the new expression levels and sequence preferences fit the data much better (data not shown).

Returning to the Wold data, we note from Table 3 that the fold change for SSP for exons is 1.12, which suggests the possibility that there may be enough differences in the exon-level estimates between the MART model and the uniform model. To assess the performance of the two models with regard to exon-level estimates, we compared our estimates of the isoform expression levels with those given in Pan *et al. *[29], who studied 3,126 'cassette-type' alternative splicing (AS) events in 10 mouse tissues using custom microarrays. Every AS event in each tissue was targeted by seven probes, and then a percent alternatively spliced exon exclusion value (%ASex) was computed as a summary statistic. In the paper by Jiang *et al. *[13], which introduced their method for estimating isoform expression levels, they compared %ASex by Pan *et al. *[29] with %ASex calculated based on the uniform model for three mouse tissues: liver, muscle and brain. In particular, they selected subsets of the AS events based on two criteria: one requires a moderate expression level of the gene and a relatively narrow confidence interval of the %ASex; and the other additionally requires a moderate percentage of the exon-excluded isoform. We used the same subsets of genes, taking the sequencing preferences predicted by MART into account, and used their approach to calculate %ASex. The results are summarized in Table 5. For almost every subset of genes, the Pearson's correlation coefficients are higher when we consider sequencing preferences, and the average relative improvement is about 7.2%. This suggests that our MART model offers meaningful improvement for the isoform expression level estimate even for the Wold data, which has the least amount of non-uniformity.

**Table 5:** Pearson's correlation coefficients of %A Sex

Selection criterion	Tissue	Number of selected AS events	PCC by uniform model	PCC by our MART model	Relative improvement
1	Liver	472	0.48	0.50	4.2%
	Muscle	451	0.40	0.45	12.5%
	Brain	699	0.36	0.40	11.1%
					
2	Liver	228	0.60	0.60	0%
	Muscle	194	0.48	0.51	6.3%
	Brain	298	0.44	0.50	13.6%

PCC: Pearson's correlation coefficient.

In the above, we find that the main factor determining how much improvement our model can bring is the magnitude of fold changes. Thus, we expect that our method can be applied to many other problems that involve short sequence elements. In new isoform discovery, a problem of great current interest, it is crucial to take into account the relative counts of reads along the region. For example, a region with more reads per base than its surrounding regions suggests a new exon. However, this might be misleading if this region has more reads merely because it has larger sequencing preferences than its surrounding regions. Further effort is needed to incorporate our method into current isoform-discovery algorithms.

While the MART model gives better estimates of sequencing preferences and is thus used for statistical inference, the main purpose of the Poisson linear model is to select a proper *K *for the MART model. Nevertheless, it might still be possible for us to get more information from it, especially from the plot of the coefficients (like Figure 2). For example, if the coefficients in the central part of the curve have large absolute values, this may indicate that the difference in sequencing preferences is repeatedly enlarged in the experiment, most likely by multi-round PCR, and we may need to use more mRNA samples instead of doing PCR for too many rounds. As another example, if the coefficient curve has heavy tails, this should indicate that the mRNA/cDNA tend to form complex non-local secondary structure, which is also unfavorable, and we may need to fragment the mRNAs into smaller pieces and/or choose better linkers with proper lengths. It might be possible for experienced technicians, who know all the details of the experiments, to provide more explanation of, or even pinpoint, the main causes of biases. This might help to improve the protocols of RNA-Seq.

## Conclusions

### Non-uniformity is dramatic in RNA-Seq data

In each of the eight sub-datasets, the RNA-Seq count data are largely over-dispersed. This is strong evidence that the non-uniformity of the counts is too great for Poisson distribution with constant rate tocapture. Also, among the sub-datasets of each dataset, the trends that counts differ along the gene show a highly consistent pattern. This is not only evidence that the Poisson distribution fails, but also suggests that the changes of the counts depend on the position along the gene.

### Poisson linear model

We proposed a Poisson linear model for the count data, and implement an iterative Poisson linear regression procedure to fit it. Using the surrounding 80 nucleotides, it is able to explain 37 to 52% of the difference in the counts for the most highly expressed genes. We find that the coefficients for nucleotides near the first nucleotide of a read have bigger abstract values, indicating that they play a more important role in determining the sequencing preferences.

### MART model

To capture the nonlinear effects of the local sequences, we use MART to fit the log preferences, and a cross-validation strategy is implemented to calculate *R*^2^. MART gives a cross-validation *R*^2 ^of 0.52 to 0.70 in seven out of eight sub-datasets, a 0.10 to 0.20 improvement. This result indicates that the major information about non-uniformity is in the local sequences.

### Benefits of our models

Our models may help us to evaluate the protocol for RNA-Seq experiments. It can also give us better estimators for the quantitative inferences of RNA-Seq data. Since the average preferences can vary substantially in short pieces of sequences, the improvement can be significant. We believe that all quantitative analysis of RNA-Seq data should incorporate sequencing preference information. Particularly, we suggest training a model for sequencing preference using only the top 100 genes and MART, then using this trained model to predict the sequencing preference of all sites in the transcriptome, which are then used in further inferences.

## Materials and methods

### Extracting the count data from the original reads data

First, we downloaded from the UCSC genome browser website [30] the sequences of RefSeq genes [31,32] (mouse July 2007 mm9 for the Wold and Grimmond data, and human Feb 2009 hg19 for the Burge data). Then, we mapped the reads to all isoforms of the RefSeq genes. For Illumina data, we directly mapped the 25 or 32 nucleotide reads using SeqMap [33], allowing two mismatches. For ABI data, we used the same strategy as described in Supplementary Figure 1 of [12], where a three-round mapping for 35, 30 and 25 nucleotide qualified reads was performed separately. In each round, we used SOCS [34] as the mapping tool. After mapping, we selected genes that have only one isoform annotated in RefSeq and do not overlap with other genes, and called them 'non-overlapped single-isoform genes'. To avoid ambiguity, we only retained reads that map to a unique site and this site is within the unique genes. Then, we counted the number of reads whose mapping starts at each position of these unique genes, which gives the count data. Since some positions have the same local sequence (to the length of reads) as other positions because of the short length of reads, they are always assigned a zero count by our counting method. This might influence the results of our analysis. However, these positions comprise less than 2% of all positions even if the read length is only 25, so they should not change our analysis significantly.

Several more steps are performed afterwards. To avoid UTR ambiguity in the annotation and boundary bias in the sequencing [3], we truncated all UTRs and a further 100 nucleotides on both ends. We then discarded genes that are too short (less than 100 nucleotides) after the truncation. Finally, after calculating the gene expression levels measured by RPKM [7], we discarded all genes except the top 100 with the highest expression levels. The counts of these top genes were the only counts we used for fitting the models. Reads from these top genes make up a considerable proportion of all reads mapped unambiguously, and thus give sufficient information for the sequencing preference. In contrast, lowly expressed genes have no or only a few reads across them, and moderate-expressed genes often have zero counts for a considerable proportion of sites; thus, information on their sequencing preference is limited.

The count data for the top 100 genes in each sub-dataset are available in the R package 'mseq' [35], which is publicly available in CRAN (The Comprehensive R Archive Network).

### Fitting the Poisson linear model

We use the following strategy to fit our Poisson regression model:

1. Initialize , where *L*_*i *_is the length of gene *i*.

2. Viewing  as known offsets, fit the Poisson regression model to get  and . This is a standard algorithm, and 'glm()' of R [26] implements it.

3. Update , where *W*_*i *_is the sum of sequencing preferences of all nucleotides of gene *i*, that is, .

4. Jump to step 2 unless the deviance decreases less than 1%.

In the above, step 2 gives the maximum likelihood estimate of *α *and *β*_*kh *_given , and it is easy to prove that step 3 gives the maximum likelihood estimate of *ν*_*i *_given *α *=  and *β*_*kh *_= . So the above procedure maximizes the likelihood by iteratively optimizing the preference parameters and the gene expression levels.

The R codes implementing this procedure are available in the R package 'mseq' [35].

### Strategy for using MART and estimating cross-validation *R*^2^

The strategy for using MART and estimating cross-validation *R*^2^ includes the following steps: (1) Randomly divide the 100 genes into 5 groups. In each fold of cross-validation, use one of them as the testing set, and the other four as the training set. (2) In each fold, for each gene in the training dataset, divide each count by the mean of counts in this gene. The resulting number is considered to be the sequencing preference of that position. To avoid zero preference, which is troublesome in step 3, we replace zero counts by a small number (0.5 in our calculation). (3) Get the logarithm of these preferences. (4) Train MART using the surrounding sequences as input and these log preferences as output. The parameters we used for MART are: interaction depth = 10, shrinkage = 0.06, and number of trees = 2000 (the method is robust to the choice of parameters; Additional file 1). Also, we put heavier weights on log preferences from more highly expressed genes since they have smaller variance. The weights for log preferences from gene *i *are set to be *N*_*i*_/*L*_*i*_, where *N*_*i *_is the total number of reads across this gene, and *L*_*i *_is the length of this gene. (5) Use the trained MART to predict the log preferences of the testing data. (6) Get the maximum likelihood estimate of the gene expression levels. That is, suppose for a gene the length is *L*, the log preferences are *a*1, ..., *a*_*L*_, and the counts are *n*_1_, ..., *n*_*L*_, then the gene expression level is

 (7) Calculate the deviance according to the log preferences in step 5 and the gene expression levels in step 6. Also calculate the null deviance. (8) Repeat steps 2 to 7 for all five folds. (9) Calculate the final cross-validation *R*^2^, which is the sum of deviances in the five folds over the sum of null deviances.

The R codes implementing this procedure are available in the R package 'mseq' [35].

## Abbreviations

ABI: Applied Biosystems; *Apoe: *apolipoprotein E; AS: alternative splicing; %ASex: percent alternatively spliced exon exclusion; bp: base pair; EB: embryoid body; ES: embryonic stem cell; MART: multiple additive regression trees; RPKM: reads per kilobase of exon per million mapped sequence reads; SSP: sum of sequencing preferences; UTR: untranslated region.

## Authors' contributions

JL, HJ and WHW conceived the study. JL developed the methods, performed the analysis, and drafted the manuscript. HJ and WHW reviewed and revised the manuscript. All authors have read and approved the final manuscript.

## Supplementary Material

Additional file 1 **Supplementary material**. Word document containing supplementary material for this paper, which provides details and discussion about the methods we propose. Click here for file
