# Supplementary material for: Modeling non-uniformity in short-read rates in RNA-Seq data
Source: Genome Biol. 2010 May 11;11(5):R50. doi: 10.1186/gb-2010-11-5-r50 (PMC2898062; doi:10.1186/gb-2010-11-5-r50)
Supplement: Additional file 1 — Supplementary material. Word document containing supplementary material for this paper, which provides details and discussion about the methods we propose. [file gb-2010-11-5-r50-S1.doc]

# Supplementary Material

## The influence of sequencing depth

The sequencing depth has an important impact on the proportion of variance that our models can explain. We use the Wold liver data as an example. In the top 100 genes, the average sequencing depth is 44, that is, each position has 44 reads whose mappings starting with it. To evaluate the impact of sequencing depth, we randomly sampled the reads so that the sequencing depth becomes 20, 10, 5, 1 or 0.1, and then applied our two methods on them. Showing below are the cross-validation $R^{2}$ curves, blue for the Poisson linear model and red for the MART model.


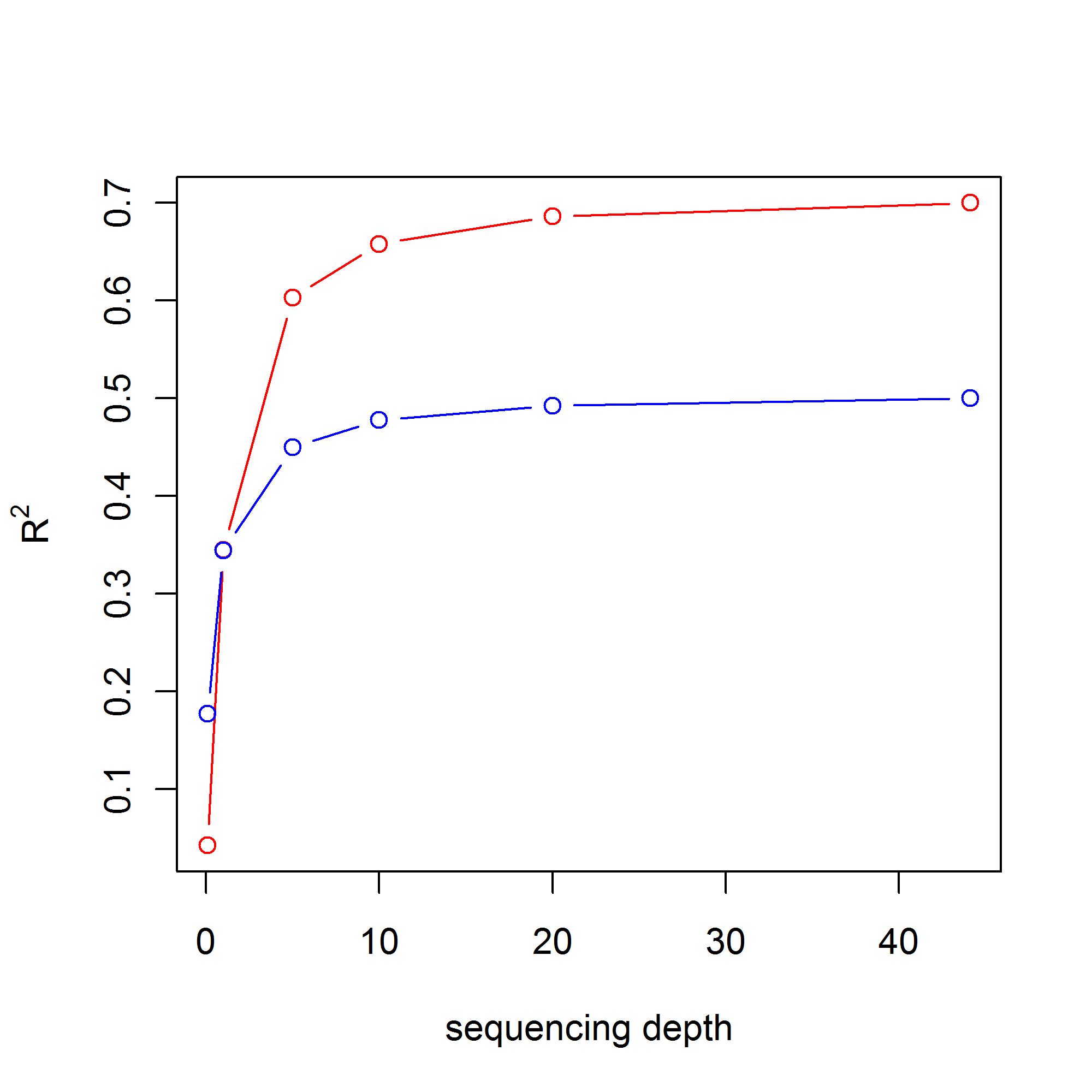


We find that while $R^{2}$ increases as the sequencing becomes deeper, it changes little when sequencing depth is over 10. This “asymptotic” $R^{2}$ shows the percentage of variance that can be explained by the model given sufficient data, which should be a good measure for the performance of the model.

## Why only the top 100 genes are used for training

The main reason that the top 100 genes are selected is that they have the highest signal-to-noise ratio (SNR). For a Poisson distribution with mean $\mu$, its standard deviation is $\sqrt{\mu}$, and hence $\text{SNR}=\text{mean}/\text{s.d.}=\sqrt{\mu}$. Counts in the lowly-expressed genes have small means and hence small SNRs. On these genes, even if we develop a “perfect” method which can predict the true means exactly, we will still get a low $R^{2}$. This places difficulty in evaluating the performance of methods. The previous section “the influence of sequencing depth” actually shows this idea clearly. In the top 100 genes, the sequencing depth is more than 10, thus there the data can be regarded as sufficient, which is not the case for most of the other genes. Why do not we use all genes with sequencing depth more than 10? Because the high cross-validation $R^{2}$ in the top 100 genes shows that the information in the top 100 genes is enough to train a good model, and thus we do not need to include more genes to lengthen the computational time.

## GC content in the top 100 genes

Since our only criterion for selecting the top 100 genes for training is the density of reads, it is unlikely that these genes belong to a distinct GC-content category. We calculated the GC contents in the top 100 genes and in all the single-transcript genes in the Wold liver data. The histograms of the GC contents are shown below. We did not find much difference between them.


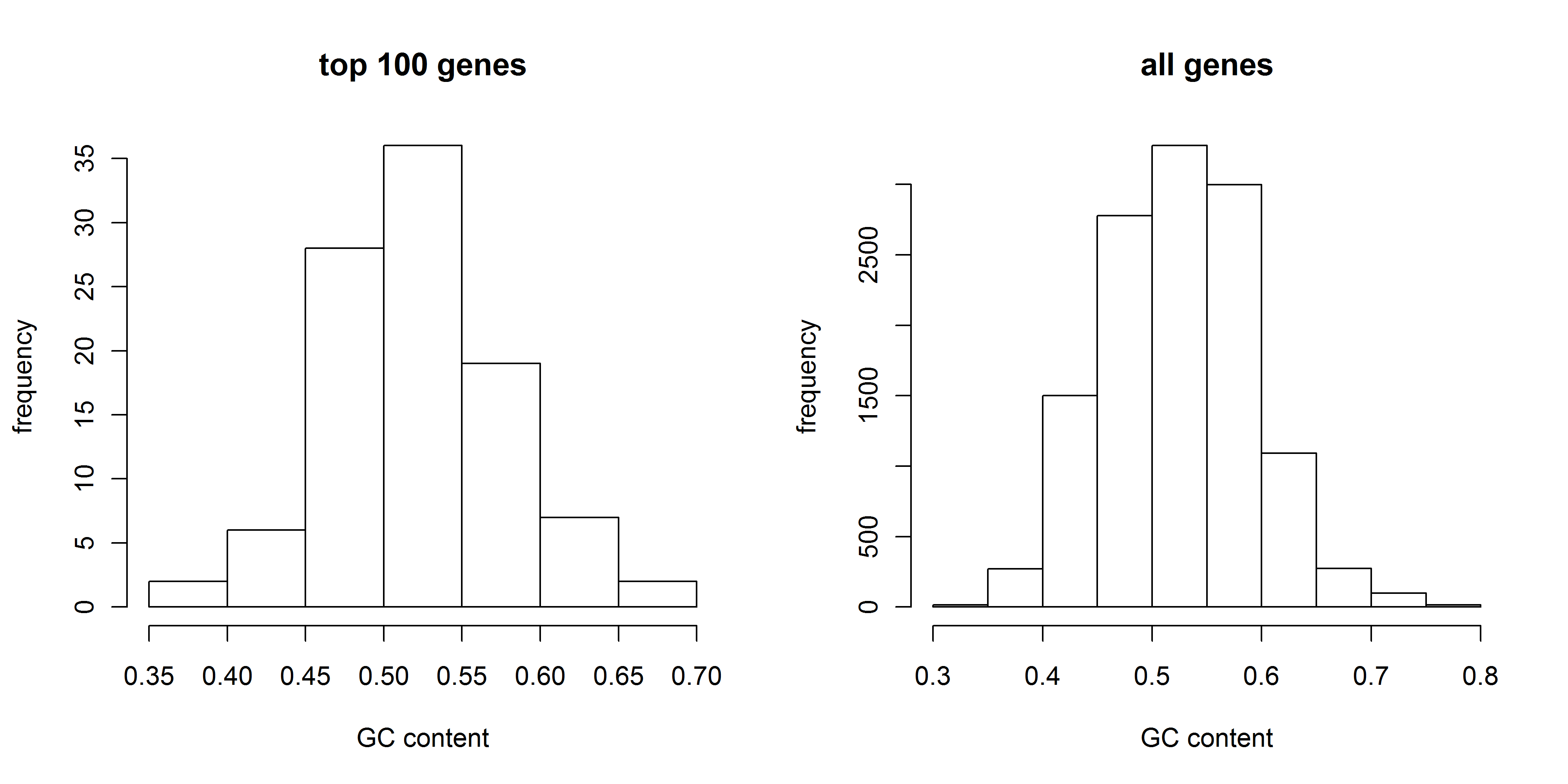


## Why choose 80-nucleotide surrounding sequences initially

Our aim is to select a large-enough window to cover the informative area. Unfortunately there is no prior knowledge about how to do this, so we select 80 nucleotides just because it seems to be large enough. Plotting the coefficients in the 80-nucleotide window, we find that the coefficients are larger in the central 40- nucleotide region, so we choose these 40 nucleotides to train our MART model, and get a high $R^{2}$. Any choice of the original window size should be considered successful if only it gives us this most informative 40-nucleotide region.

When we change the original window size, we find that the coefficients in the central part changes little, and the coefficients in both tails remain small. In the following figure, we show the coefficients for original windows of 120 nucleotides (dotted lines), 80 nucleotides (dashed lines) and 60 nucleotides (solid lines). Still, red for letter T, green for letter A, blue for letter C and black for letter G. We find that the lines for the same letter are almost indistinguishable (in positions they overlap). So they all give us the 40-nucleotide most informative region and should be considered successful.


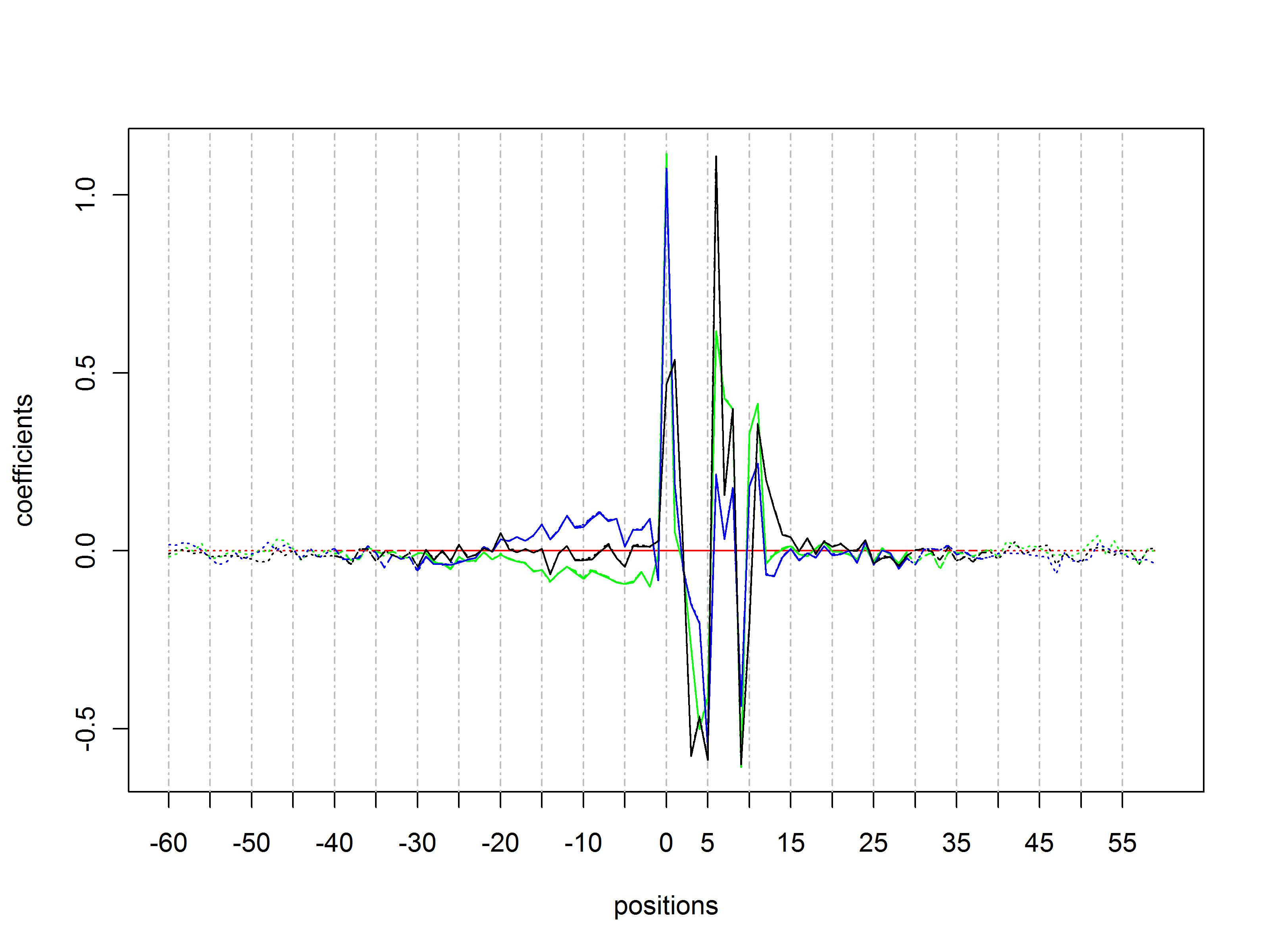


## Linear models based on GC content

Using GC content only can explain merely a small fraction of variance captured by our models. In Wold liver data, we use the occurrence of G/C in each position of the 80-nucleotide window as predictors (80 predictors totally) to train a Poisson linear model, and find that the non-cross-validation $R^{2}$ is only 0.09. Since GC contents of any regions in the 80-nucleotide window are linear combinations of these 80 predictors, regression models based on them cannot have non-cross-validation $R^{2}$ larger than 0.09. Further, since cross-validation $R^{2}$ is always no larger than non-cross-validation $R^{2}$ for any model, any regression models based on GC contents cannot have cross-validation $R^{2}$ larger than 0.09, which is only a small fraction of variance captured by our models. Our result does not contradict with the results of Dohm et al. [15], since they are considering sum of counts and GC contents in 1 kb windows, which is quite different from the base level case. Actually, it has also been shown by Mortazavi et al. [7] that simple GC content cannot explain base level sequencing bias.

## Using dinucleotide composition for Poisson linear model

Before choosing MART, we tried many ways of including more variables in the Poisson linear model, but none leads to better performance. For example, we tried to include higher order terms of the single letter configuration as Johnson et al. [17] did, or include the total times of occurrence of each of the dinucleotide compositions.

One of the anonymous reviewers suggests us to include the dinucleotide composition of each adjacent pair. Considering the case of including 40 nucleotides in our model, the original Poisson model has $40\times(4-1)=120$ parameters for the single nucleotide composition. To also include all the dinucleotide compositions, we need $(40-1)\times(4-1)\times(4-1)=351$ more parameters. This number seems to be too large but the results turn to be quite good. The cross-validated $R^{2}$ for the 8 sub-datasets (in the same order as in Table 2) are 0.65, 0.64, 0.57, 0.49, 0.43, 0.51, 0.55, and 0.52, respectively, which are still 0.02 ~ 0.06 lower than the MART model. Although the MART model should be preferred for its high performance, this linear model may still be very valuable for its simplicity. So we include its implementation in our R package “mseq” [35].

## Why choose MART

Support vector machines are much slower than MART for data of our size. Considering that we need to do cross-validation, the running time is unacceptably long. Neural network has many structural parameters to tune up, making it computationally slow and frustrating to use. On the other hand, MART is fast enough even for data of our size, and not sensitive to its parameters, which will be shown in detail in the next section. Especially, MART is a boosting-based method, which is often not likely to suffer from overfitting.

## How to choose parameters for MART

The MART model is quite robust to its parameters, which is a reason why we chose it. We use the Wold liver data as an example. The parameters we use are interaction depth = 10, number of trees = 2000, and shrinkage = 0.06, which gives (cross-validation) $R^{2}=0.70$. Changing the interaction depth to 6 or 15 does not change $R^{2}$. Changing the number of trees to 1000 gives $R^{2}=0.69$, to 3000 gives $R^{2}=0.70$. Changing the shrinkage to 0.04 or 0.10 still gives $R^{2}=0.70$. Carefully inspection and more experiments shows that generally a large number of deep trees with a small shrinkage parameter are preferred, but this choice also indicates longer calculation time, while the improvement is tiny. We believe our parameters are suitable for modeling most short-read RNA-Seq data.

## How to select *K* for MART model

We recommend selecting *K* for the MART model based on the coefficients of the Poisson model. The range where coefficients have large absolute values should be selected. This way to select *K* is intuitive rather than objective, since we assume that region with very small coefficients in Poisson model contains little information about the sequencing preferences. Fortunately, we find our criterion does work well for our data. Take the Wold liver data as an example, where we use left 25 nucleotides and right 15 nucleotides as the surrounding sequences and get $R^{2}=0.70$. If we exclude 5 nucleotides from both sides, then we get $R^{2}=0.63$, which is significantly smaller. Instead, if we include 5 nucleotides more from both sides, that is, left 30 nucleotides and right 20 nucleotides totally, we still get $R^{2}=0.70$, showing that including some more positions is not likely to improve the performance, but the computational time increases.
